# *BRAF* Mutation Is Associated with Improved Local Control of Melanoma Brain Metastases Treated with Gamma Knife Radiosurgery

**DOI:** 10.3389/fonc.2016.00107

**Published:** 2016-05-02

**Authors:** Ian S. Gallaher, Yoichi Watanabe, Todd E. DeFor, Kathryn E. Dusenbery, Chung K. Lee, Matthew A. Hunt, Hong-Yiou Lin, Jianling Yuan

**Affiliations:** ^1^Department of Radiation Oncology, University of Minnesota, Minneapolis, MN, USA; ^2^Clinical and Translational Science Institute, University of Minnesota, Minneapolis, MN, USA; ^3^Department of Neurosurgery, University of Minnesota, Minneapolis, MN, USA; ^4^Department of Radiation Oncology, Beaumont Health System, Detroit, MI, USA

**Keywords:** melanoma, brain metastases, *BRAF*, stereotactic radiosurgery, gamma knife

## Abstract

**Objectives:**

Evidence has implicated a possible role of tumor mutation status on local control (LC) with radiotherapy. *BRAF* is a proto-oncogene that is mutated in approximately 50% of patients with melanoma. We sought to analyze the influence of *BRAF* status on LC of melanoma brain metastases (MBM) following Gamma Knife radiosurgery (GK).

**Methods:**

Among 125 patients treated with GK for MBM at our institution between 2006 and 2015, we identified 19 patients with 69 evaluable metastases whose *BRAF* mutation status was known and follow-up imaging was available. LC of individual metastases was compared based on *BRAF* mutation status using statistical techniques to control for measurements of multiple metastases within each patient. CNS progression was defined as either local failure or development of new lesions.

**Results:**

Of the 69 metastases, *BRAF* was mutated in 30 and wild-type in 39. With a median follow-up of 30 months for all patients and a median follow-up of 5.5 months for treated lesions, 1-year LC was significantly better among metastases with mutated vs. wild-type *BRAF* (69 vs. 34%, RR = 0.3, 95% CI = 0.1–0.7, *p* = 0.01). *BRAF* mutation was found to be a significant predictor of LC after stereotactic radiosurgery (SRS) in both univariate [RR = 0.3 (95% CI 0.1–0.7, *p* = 0.01)] and multivariate [RR = 0.2 (95% CI 0.1–0.7, *p* = 0.01)] analyses. There was also a trend toward improved CNS progression free survival (PFS) at 1 year (26 vs. 0%, *p* = 0.06), favoring *BRAF*-mutated patients.

**Conclusion:**

In this retrospective study, MBM treated with GK had significantly improved LC for patients with *BRAF* mutation vs. wild-type. Our data suggest that *BRAF* mutation may sensitize tumors to radiosurgery, and that *BRAF* wild-type tumors may be more radioresistant.

## Introduction

Melanoma is the fifth most common malignancy in the US, but the third most common cause of brain metastases. Of the estimated 75,000 people diagnosed annually with melanoma, 7.4–10% will develop brain metastases (1–3). Among patients presenting with stage IV melanoma, over 40% have or will develop brain metastases (4). Patients with melanoma brain metastases (MBM) have a 95% chance of dying from their intracranial disease with a median survival time of 6.7 months (3, 5). Treatment for MBM includes surgical resection, stereotactic radiosurgery (SRS), whole brain radiation therapy (WBRT), steroids, or a combination of these. In patients with few, asymptomatic metastases, initial treatment with SRS alone is preferred (6–8). However, approximately 30% of MBM fail locally after SRS (9–11).

Response to SRS for brain metastases depends on several factors. Tumor histology, size, and marginal dose are well-established predictors of local control (LC) (9–13). Emerging evidence suggests that the mutation status within a particular tumor may also influence LC after SRS. Johung et al. analyzed non-small cell lung cancer (NSCLC) patients with brain metastases treated with SRS and found that tumors with *EGFR* mutations or *EML4–ALK* translocations rarely recurred in-field, whereas those that lacked such mutations or harbored *KRAS* mutations were more likely to experience in-field recurrence (14). Other researchers also reported higher response rates to brain radiation among NSCLC patients with mutant *EGFR* compared to those with wild-type *EGFR* (15). *EGFR* and *ALK* mutations result in constitutive activation of the MAPK pathway, which may confer radiosensitivity (16).

Mutations in the *BRAF* gene have been reported in approximately 50% of the patients with melanoma (17, 18). The most common mutation, *BRAF-V600E*, results in substitution of the valine residue with glutamic acid, locking the kinase into a constitutively active conformation. Because BRAF is also an important protein in the MAPK pathway, we hypothesized that *BRAF* mutation status may influence LC of MBM treated with SRS. We, therefore, identified melanoma patients treated with SRS at our institution and analyzed their outcomes based on *BRAF* mutation status.

## Materials and Methods

### Patients

From a database of patients treated with Gamma Knife (GK) SRS at our institution, we identified patients treated for MBM. Patients were included in this study if their *BRAF* mutation was known and at least one posttreatment imaging follow-up was available. WBRT, given either before or after SRS, was allowed, however, tumors previously treated with GK or surgical resection were excluded. This study was approved by the institutional review board (IRB code number: 1501M60361).

### BRAF Mutation

*BRAF* mutation was tested on tissue obtained from biopsy or resection of extra-cranial melanoma. Patients with mutant *BRAF* were eligible for treatment with BRAF inhibition. BRAF inhibition was given at the discretion of the medical oncologist. The majority of patients were treated with vemurafenib. One patient also received dabrafenib.

### Radiation Treatment

Gamma Knife stereotactic radiosurgery was performed using the Leksell Gamma Knife Model 4C (Elekta AB, Stockholm, Sweden). Radiation dose was selected, with modifications by the prescribing physician, based on tumor size according to the RTOG 9005 trial (19). In general, tumors measuring <2, 2–3, and 3–4 cm received 24, 18, and 15 Gy, respectively, with doses generally prescribed to the 50% isodose line. WBRT was delivered from 6-MV parallel-opposed beams at 30 Gy in 10 (*n* = 7) or 20 Gy in five fractions (*n* = 1).

### Follow-up and Endpoints

After GK SRS, patients were followed at 3-month intervals with magnetic resonance imaging (MRI) performed at each visit. Patient follow-up duration was defined as time from first GK to last imaging visit or death.

Tumor size was measured in three dimensions on axial, coronal, and sagittal views of pretreatment and follow-up gadolinium-enhanced T1 phase MRI. Tumor volume was estimated with the ellipsoid volume formula. The accuracy of this technique has previously been verified with respect to volume as measured by Leksell Gamma Plan (9). LC of individual metastases was based on modified RECIST criteria. A tumor was defined as locally failed if there was a relative increase in tumor volume on follow-up MRI by ≥20% compared to pretreatment MRI (9, 20). Necrosis or hemorrhage was included in volume quantification unless clearly present outside of the tumor. Lesions (*n* = 3) that returned to <20% of pretreatment MRI volume on subsequent imaging with only conservative management were considered controlled. The transient volume increase was interpreted as tumor swelling and/or necrosis rather than treatment failure. CNS progression was defined for patients as either local failure of treated lesions or development of new lesions in the brain.

### Statistics

Patient demographic and treatment characteristics were compared based on *BRAF* mutation status using the Chi-square test or Fisher’s Exact test for categorical variables based on expected cell counts. The general Wilcoxon test was used for continuous variables. Follow-up was measured by reverse Kaplan–Meier curves. CNS progression-free survival (PFS) and overall survival (OS) were calculated from the date of first GK to the date of progression or death, and were estimated with Kaplan–Meier curves. Comparisons were completed with the log-rank-test.

Local control was analyzed for individual metastases using two methods, both of which accounted for assessment of multiple metastases within each patient. First, crude LC at 3 and 6 months was estimated using a non-linear mixed logistic regression. The correlation of clustering of metastases within patients was modeled assuming a normally distributed random variance component (21). All metastases were evaluable for the 3-month assessment. Fifteen metastases were excluded from the 6-month assessment due to lack of imaging. Second, LC was estimated with Kaplan–Meier curves and compared with a Frailty–Cox model accounting for within-cluster correlation by incorporating cluster effects as independent and identically distributed random variables (22). LC was censored for early patient death or loss of follow-up. Although patient numbers were small, we attempted to control for potential confounding of the effect of *BRAF* mutation on the risk of failure by use of multiple regression in the Frailty–Cox model. Additional variables included in the model were use of WBRT, marginal dose (<22 vs. ≥22 Gy), and maximum tumor dimension (<0.80 vs. ≥0.80 cm). Treatment volume was excluded from the model due to its high correlation with tumor dimension. All tests were two-sided and considered significant if *p* < 0.05. SAS 9.3 (SAS Institute, Cary, NC, USA) and R 3.0.2 (R foundation for Statistical Computing, Vienna, Austria) were used to perform all statistical analyses.

## Results

### Patient Characteristics

We identified 125 patients treated with GK for MBM at our institution since 2006. Of these, 104 patients had either unknown *BRAF* status (routine testing began in 2011) or inadequate follow-up imaging, and therefore, were excluded, leaving 19 eligible patients. Of these patients, *BRAF* was mutated in 11 and wild-type in 8. Characteristics of these two groups of patients were similar with respect to age, gender, presence of extracranial metastases, GPA, number of metastases, use of WBRT and follow-up duration (Table 1). Of the 11 patients with mutant *BRAF*, all but one received a BRAF inhibitor at some point in their treatment course.

**Table 1:** **Characteristics of patients by *BRAF* status**.

Variable	*BRAF* wild-type (*N* = 8)	*BRAF*-mutated (*N* = 11)	*p*-value^a^
Median, age, and range	58 (42–73)	51 (32–78)	0.32
Gender: male	4 (50%)	5 (45%)	0.99
Presence of extra-cranial metastases	8 (100%)	9 (82%)	0.49
GPA			0.30
1	0 (0%)	2 (18%)	
2	3 (38%)	2 (18%)	
3	5 (63%)	4 (36%)	
4	0 (0%)	3 (27%)	
KPS			0.36
<70	0 (0%)	1 (9%)	
70–80	4 (50%)	3 (27%)	
90–100	4 (50%)	7 (63%)	
Number of metastases at first GK			0.99
1	3 (38%)	5 (45%)	
2	2 (25%)	2 (18%)	
≥3	3 (38%)	4 (36%)	
Median	2 (1–9)	2 (1–50)	
Use of WBRT: yes	4 (50%)	4 (36%)	0.37
Median F/U (months)	26 (9–26)	32 (25–33)	0.87

*^a^*p*-value for between-treatment comparisons. Continuous variables were analyzed by general Wilcoxon test. Categorical variables were analyzed by Fisher’s exact test*.

### Analysis of Individual Metastases

Among the 19 eligible patients, a total of 69 tumors were treated with GK SRS and had adequate follow-up imaging. Of these, 39 were from patients with wild-type *BRAF* and 30 were from patients with mutated *BRAF*. Characteristics of these metastases were compared based on *BRAF* status. Metastases from patients with wild-type *BRAF* were more likely to have been exposed to WBRT therapy before or after GK (79 vs. 43%, *p* < 0.01). Otherwise, there were no significant differences between the two groups (Table 2). The median treatment volume of individual tumors for wild-type vs. mutated *BRAF* was 2.1 mL (0.2–284.8 cc) vs. 0.8 mL (0.1–99.6 mL), *p* = 0.45. The maximum tumor dimension was 1.0 cm (0.2–6.2 cm) vs. 0.8 cm (0.3–2.9 cm), *p* = 0.15, respectively. Median marginal dose was 22 Gy (12–24 Gy) for both groups, commonly prescribed to the 50% isodose line. Median follow-up from GK to last follow-up MRI for individual tumor was 5.5 months (range = 0.7–29.8 months). Of the 30 metastases with mutated *BRAF*, all but one metastasis were exposed to a BRAF inhibitor at some point in the treatment course. Regarding the timing of BRAF inhibitor treatment in relation to GK SRS, 2 were exposed before GK (by at least 13 days); 10 were exposed before, during, and after; 6 were exposed both before (>18 days) and after (>6 days), but held during GK SRS; and 11 were exposed after (>10 days).

**Table 2:** **Characteristics of metastases by *BRAF* status**.

	*BRAF* wild-type (*N* = 39)	*BRAF*-mutated (*N* = 30)	*p*-value^a^
Use of WBRT	31 (79%)	13 (43%)	<0.01
GK as initial treatment	14 (36%)	3 (10%)	0.01
GK as salvage treatment after WBRT	17 (44%)	10 (33%)	0.39
Median treatment volume (mL)	2.1 (0.1–284.8)	0.8 (0.1–99.6)	0.45
Median max treatment dimension (cm)	1.0 (0.2–6.2)	0.8 (0.3–2.9)	0.15
Median marginal dose (Gy)	22 (15–24)	22 (12–24)	0.63

*^a^*p*-value for between-treatment comparisons. Continuous variables were analyzed by general Wilcoxon test. Categorical variables were analyzed by the Chi-square test*.

**Figure 1:**
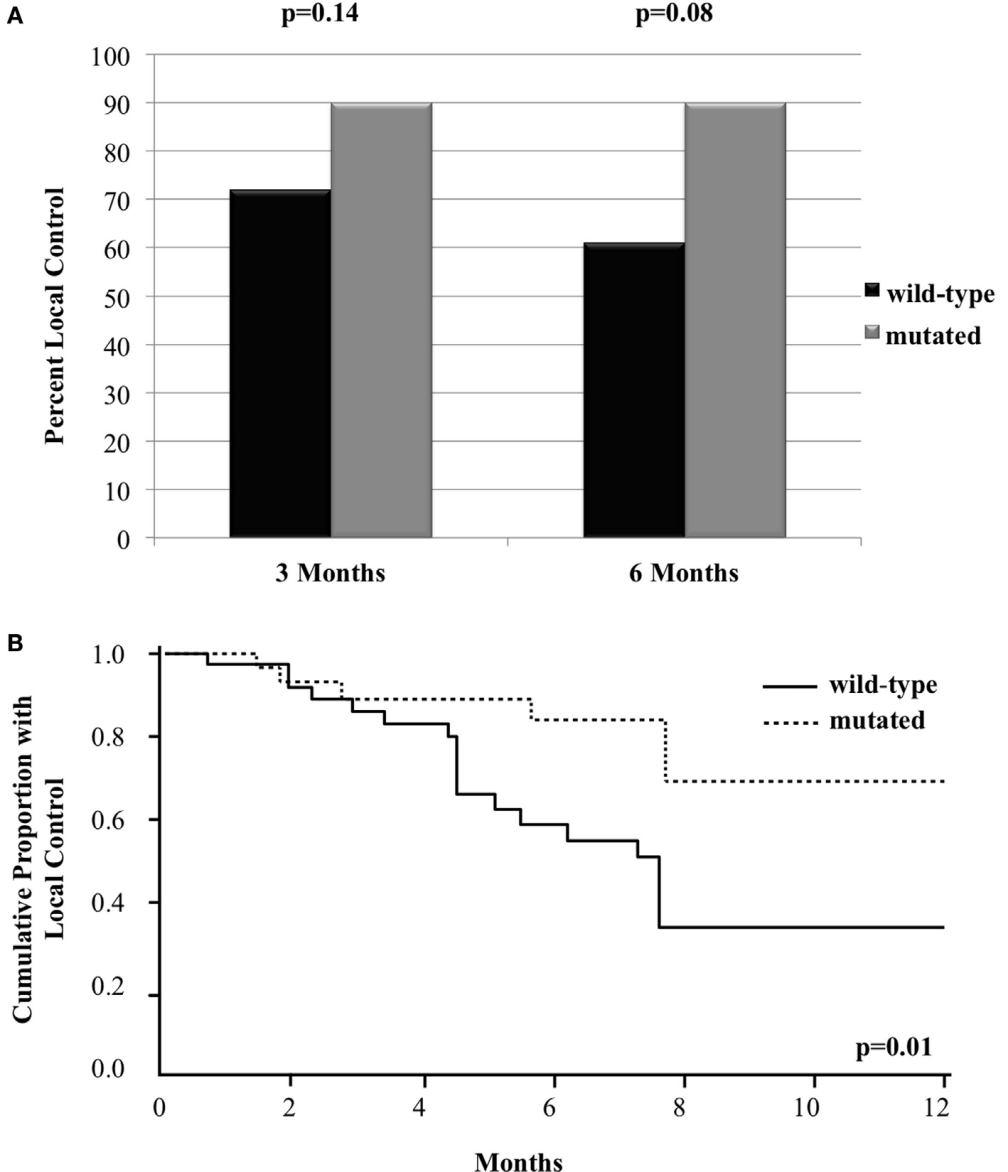
**Local control of metastases in patients with mutated compared to wild-type *BRAF* with (A) bar graph of crude local control at 3 and 6 months and (B) Kaplan–Meier curve of overall local control through 12 months**.

Of the 69 metastases treated with GK, 41 achieved LC (59%) at a median follow-up of 5.5 months. The 3- and 6-month crude LC for all the treated tumors was 80 and 72%, respectively. When analyzed based on *BRAF* status, there was a trend for improved crude LC among metastases with mutated *BRAF* as follow-up time increased. At 3 and 6 months following treatment, crude LC was 72 vs. 90% (*n* = 69, *p* = 0.14) and 61 vs. 90% (*n* = 54, *p* = 0.08), respectively (Figure 1A). Since there was loss of information in assessing LC beyond 6 months using mixed models, we also assessed LC with a Frailty model through 1 year, which can incorporate censored patients. With this method, there was a statistically significant improvement in LC among metastases with mutated *BRAF*. At 1 year, LC was 34 vs. 69% [RR = 0.3 (95% CI 0.1–0.7, *p* = 0.01)] for *BRAF* wild-type vs. *BRAF* mutant metastases, respectively (Figure 1B).

### Univariate and Multivariate Analysis

We analyzed the factors affecting LC using univariate analysis (Table 3). In addition to *BRAF* mutation status being predictive of better LC, a higher marginal dose of ≥22 Gy was also associated with improved LC (67 vs. 27% at 1 year, *p* = 0.03). On multiple regression analysis (Table 4), *BRAF* mutation remained an independent predictor of LC [RR = 0.2 (95% CI 0.1–0.7, *p* = 0.01)]. The *p*-value for the effect of marginal dose did not reach significance. LC was not influenced by max tumor dimension or the use of WBRT. Because all but one metastasis with mutated *BRAF* were exposed to a BRAF inhibitor, analysis of LC with regard to exposure of targeted therapy was not conducted. However, we did analyze the timing of BRAF inhibitor exposure relative to GK SRS and found no correlation with LC among the 29 metastases that received targeted therapy (Table 5).

**Table 3:** **Univariate analysis of local control for metastases treated with GK SRS**.

Factor	*N*	# events	Local control at 1 year (%)	Median months to failure	Relative risk of failure (95% CI)	*p*-value
Overall	69	26	49	7.7		
*BRAF*						0.01
Wild-type	39	19	34	4.5	1.0	
Mutated	30	7	69	5.6	0.3 (0.1–0.7)	
WBRT						0.19
No	25	7	65	2.7	1.0	
Yes	44	19	40	6.2	1.9 (0.7–5.2)	
Treatment volume (ml)						0.83
<1.0	34	14	52	7.4	1.0	
≥1.0	35	12	44	4.5	1.1 (0.5–2.5)	
Max tumor dimension (cm)						0.46
<0.80	29	16	55	7.6	1.0	
≥0.80	40	10	45	4.5	1.4 (0.6–3.2)	
Marginal dose						0.03
<22 Gy	30	16	27	5.3	1.0	
≥22 Gy	39	10	67	3.9	0.4 (0.2–0.9)	

**Table 4:** **Multiple regression analysis of local control for metastases treated with GK SRS**.

Factor^a^	*N*	# events	Relative risk of failure (95% CI)	*p*-value
*BRAF*			0.01	0.01
Wild-type	39	19	1.0	
Mutated	30	7	0.2 (0.1–0.7)	
WBRT				0.92
No	25	7	1.0	
Yes	44	19	0.9 (0.3–3.0)
Marginal dose				0.07
<22 Gy	30	16	1.0	
≥22 Gy	39	10	0.4 (0.2–1.1)	
Max tumor dimension				0.89
<0.80 cm	29	16	1.0	
≥0.80 cm	40	10	0.9 (0.3–2.5)	

*^a^Tumor volume not included due to high correlation with tumor dimension*.

**Table 5:** **Univariate analysis of timing of BRAF inhibitor on local control for metastases treated with GK SRS**.

Timing of BRAF inhibitor use	*N*	# events	Local control at 1 year (%)	Median months to failure	Relative risk of failure (95% CI)	*p*-value
After GK SRS	11	4	64	7.7	1.0	0.71
Before and after GK SRS	16	2	87	1.6	2.0 (0.2–17.8)	
Before GK SRS	2	1	50	2.7	0.7 (0.1–4.0)	

### Patient Outcomes

**Figure 2:**
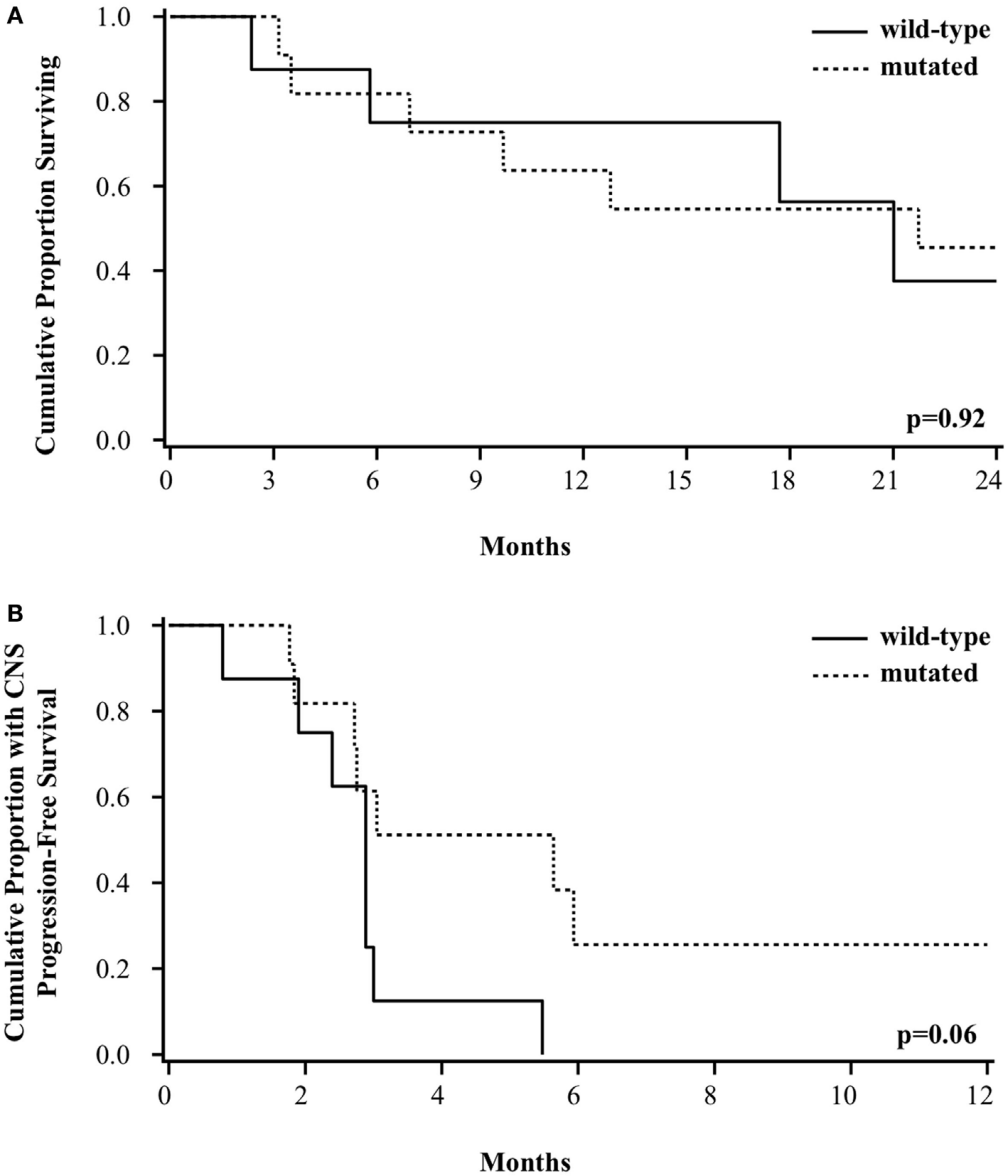
**Kaplan–Meier curve of (A) overall survival and (B) CNS progression-free survival among patients based on *BRAF* mutation status**.

At a median follow-up of 30 months, the median OS for the entire group of patients was 21 months. Survival was not different based on *BRAF* mutation status. The median OS was 21 months for patients with wild-type or mutated *BRAF*, with a 2-year survival of 38% (95% CI 6–72%) vs. 45% (95% CI 17–71%), respectively (*p* = 0.92, Figure 2A). There was a trend toward improved CNS PFS among patients with *BRAF* mutation [26% (95% CI 4–56%) vs. 0% at 1 year, *p* = 0.06, Figure 2B]. The incidence of CNS progression for wild-type vs. mutated *BRAF* was 87% (95% CI 58–99%) vs. 39% (95% CI 16–73%), respectively at 3 months (*p* = 0.11) and 100 vs. 74%, respectively, at both 6 and 12 months (*p* = 0.06). The improved CNS PFS seen among the *BRAF-*mutated patients appears to be primarily attributed to a better LC of GK-treated metastases. Of the 8 patients with wild-type *BRAF*, all patients failed at at least 1 treated local sites (2 local only, 6 local and distant), whereas of the 11 patients with mutated *BRAF*, only 5 patients experienced local failure (all of whom also had distant failure).

## Discussion

In this retrospective study, we examined the LC of MBM treated with GK SRS with respect to *BRAF* mutation status. We found a significantly improved LC among metastases carrying *BRAF* mutation compared to those with wild-type *BRAF*. Among brain metastases harboring mutated *BRAF*, 1-year LC was 69%, whereas those with wild-type *BRAF* have an inferior LC of 34% (*p* = 0.01). There was also a trend toward improved CNS PFS among patients with mutated *BRAF*, which appears to be primarily due to better LC of GK-treated metastases.

Our finding that the presence of *BRAF* mutation is associated with an improved LC raises the possibility that *BRAF* mutation may directly influence radiosensitivity when these tumors are treated with SRS. This is in keeping with recent observation made in NSCLC, in which patients with *EGFR* mutations or *EML4–ALK* translocations experience a significantly better in-field control compared to those without such mutations or those harboring *KRAS* mutations instead (14, 15). These mutations are similar to *BRAF* mutation in that they all result in constitutive activation of the MAPK pathway (16). The association could suggest a common mechanism such as increased proliferative rate and thus increased susceptibility to radiation therapy.

To our knowledge, there is only one published study that also addressed the influence of *BRAF* mutation status on LC following SRS for MBM (23). The study included 52 melanoma patients with 185 treated brain metastases. Unlike our study, a difference in LC with regard to mutation status was not found. The 1-year LC was 67.1% among MBM in patients with wild-type *BRAF* and 70.0% for MBM in patients with *BRAF* mutation. Instead, they found that the use of BRAF inhibitor was significantly associated with 1-year LC (85.0% with vs. 51.5% without) (23). All but one patient with mutant *BRAF* in our study received a BRAF inhibitor at some point during their treatment course. It is possible that the improved LC observed in our patients is entirely conferred by the use of a BRAF inhibitor.

Several other studies reported the use of BRAF inhibitors in the setting of brain radiation therapy. In a small series by Narayana et al., 12 patients with *BRAF* mutations were treated with either SRS or WBRT prior to or along with vemurafenib. A 75% radiographic response was observed among index lesions, including a 48% complete response and a 27% partial response (24). In another study, 24 patients with 80 metastatic brain lesions were treated with SRS while on vemurafenib. LC at 6 months and 1 year was 92 and 75%, respectively and was felt to be better than reported control rates among melanoma patients treated with SRS alone. The authors suggested that there may be a synergistic effect between BRAF inhibitors and SRS (25). However, neither study compared the outcome directly to *BRAF*-mutated patients not treated with an inhibitor. Therefore, the possibility still exists that *BRAF* mutation itself may impact radiosensitivity directly.

It is not clear whether or how BRAF inhibition acts synergistically with SRS. In the aforementioned study by Ly et al., the majority of lesions were not treated with a BRAF inhibitor within a 30-day period before or after SRS. Among the remaining lesions, a washout period of 7 days (range 1–20 days) was instituted (23). However, the median elimination half-life is only 50 h for vemurafenib, and even shorter for dabrafenib (5 h) (26, 27). It is therefore difficult to account for the improved LC observed using a traditional radiosensitization model in which the drug is expected to be present at the time of radiation, as shown *in vitro* for melanoma cell lines (28, 29). We also analyzed the timing of BRAF inhibitor exposure on LC with SRS, and we did not observe any correlation. However, our sample size was small with only 29 metastases receiving targeted therapy.

Another unresolved issue is in regard to the safety of the concurrent use of a BRAF inhibitor with SRS. An increased rate and severity of radiation dermatitis has been observed when BRAF inhibition is used concomitantly with conventionally fractionated radiotherapy. This has lead many investigators to transiently hold drug therapy for patients undergoing SRS. However, the necessity and appropriate duration of the “washout” period has not been established. In the study by Ly et al., the median washout period ranged from 1 to 20 days with a median of 7 days (23). In the study by Ahmed et al., patients were told to hold the drug for 2–3 days before and after SRS treatment (25). Yet in another study that primarily assessed the safety of concurrent treatment, 20 patients had no interruption of BRAF inhibitor while undergoing SRS. No cases of radiation-induced necrosis and no scalp radiation dermatitis occurred. A relatively small rate of edema and hemorrhage was detected, which is not unexpected following SRS for MBM (30). This is in contrast to the study by Ly et al. that reported a significantly increased intratumoral hemorrhage risk among patients treated with a BRAF inhibitor. Given the 1-year freedom from intratumoral hemorrhage rate of only 39.3%, the authors recommend discontinuation of BRAF inhibitors for 1–2 weeks both before and after treatment, provided systemic disease is controlled (23). We did not observe an unexpectedly high rate of posttreatment intratumoral hemorrhage among the 10 patients treated concurrently with a BRAF inhibitor. The presence of hemorrhage on MRI was not always commented upon by radiologist, and therefore, our number could be underreported. While these issues are being sorted out, it remains an attractive hypothesis that if *BRAF* mutation itself is associated with an enhanced radiosensitivity to SRS, then the use of a lower marginal dose may decrease the risk of tumor hemorrhage without compromising LC in this group of patients. This would be particularly helpful when there is concern for systemic disease progression if the BRAF inhibitor is held.

During final manuscript preparation, we came across a study similar to ours presented by Kotecha and colleagues in abstract form (31). They reported 25 patients with MBM treated with SRS. *BRAF*-mutated patients had a significantly improved OS and PFS compared to *BRAF* wild-type patients. Of the 48 MBM with recurrence information available, they found a higher rate of in-field failure among *BRAF* wild-type metastases (8/20) vs. those that are *BRAF*-mutated (4/28; *p* = 0.04). Their results, therefore, corroborate our finding, also suggesting the potential impact of *BRAF* status on outcome after SRS.

Our study has several limitations. Our sample size is small with only 69 eligible metastases in 19 eligible patients. Despite this size, there were trends that could reach significance with larger sample sizes. Another limitation is the retrospective nature of the study, which inherently does not allow for control of unknown variables between the two groups. It is encouraging that Kotecha et al. recently reported similar findings. Nevertheless, confirmatory studies from other institutions and from larger databases would be required to fully assess the impact of *BRAF* on SRS outcomes. Finally, the mutation status of *BRAF* obtained from the primary site or a more accessible metastatic site was assumed for the intracranial tumors. Metastatic tumors may change molecular subtype during disease course as has been shown for breast cancer (32).

## Conclusion

In this retrospective study, we analyzed the outcome of MBM treated with GK SRS and found that lesions with *BRAF* mutation appear to have an improved LC compared to those with wild-type BRAF. It is unclear whether this is caused by inherent radiosensitivity associated with the mutation, or due to the use of a BRAF inhibitor. Further studies will be required to fully characterize the role of *BRAF* mutation in LC of brain metastases treated with SRS.

## Author Contributions

IG and JY designed the research, collected, analyzed, interpreted the data, and wrote the manuscript. YW, MH, KD, CL, and H-YL designed the research, collected the data, and revised the manuscript. TD (statistician) analyzed and interpreted the data and drafted portions of the manuscript. All authors are accountable for all aspects of work in ensuring that questions related to accuracy or integrity of work are appropriately investigated and resolved.

## Conflict of Interest Statement

The authors declare that the research was conducted in the absence of any commercial or financial relationships that could be construed as a potential conflict of interest.
